# Effect of nipa palm (*Nypa fruticans* Wurmb.) vinegar on the incretin hormones and intestinal glucose transporters in type 2 diabetes mellitus rat model

**DOI:** 10.1186/s12906-025-04933-8

**Published:** 2025-05-30

**Authors:** Nur Izzati Razali, Tri Widyawati, Dwi Rita Anggraini, Vuanghao Lim, Nor Adlin Yusoff

**Affiliations:** 1 Department of Toxicology, Advanced Medical and Dental Institute, Universiti Sains Malaysia, Sains@Bertam, Penang, 13200 Malaysia; 2 Department of Pharmacology and Therapeutic, Faculty of Medicine, Universitas Sumatera Utara, Medan, 20155 Indonesia; 3 Department of Anatomy, Faculty of Medicine, Universitas Sumatera Utara, Medan, 20155 Indonesia

**Keywords:** Nipa palm vinegar, *Nypa fruticans* Wurmb., Incretin, SGLT1, GLUT2

## Abstract

Nipa palm vinegar has been traditionally used to lower blood glucose levels by diabetic patients. This study aims to analyse the effect of aqueous extract (AE) of nipa palm vinegar on glycemic parameters and glucose transporter-incretin hormonal system in type 2 diabetic rat. The model was established using a combination of high-fat diet (p.o.) and low dose streptozotocin (i.p.). AE (250, 500, and 1000 mg/kg) was administered orally once daily for 28 days. Biochemical parameters related to type 2 diabetes including fasting glucose, serum insulin, lipid profiles, incretin hormone, liver, and pancreatic histology were evaluated. Relative expression of jejunal glucose transporters was also determined. Induction of diabetes caused significant (*p* = 0.026) weight loss, hyperlgycemia, hypoinsulinemia, dyslipidemia and reduced incretin hormones. Diabetes onset also disturbed HOMA-IR and HOMA-ß cell function indices, altered the morphological features of hepatocytes and pancreatic islet and overexpressed intestinal glucose transporters, SGLT1 and GLUT2. Repetitive oral administration of AE (1000 mg/kg) for 28 days ameliorated the biochemical abnormalities and improved HOMA-β cell function of diabetic rats. Histological studies revealed AE treatment preserved the integrity of pancreatic islet and protected hepatocytes from degeneration and atrophic effects of streptozotocin. Further analysis suggested the effect of AE in stimulating incretin hormones secretion via the action of DPP4 inhibitor and by modulating jejunal SGLT1 expression. In conclusion, the study suggested AE exerted its antidiabetic effect partially by stimulating insulin secretion via incretin hormone and intestinal glucose transporter pathway.

**Supplementary Information:**

The online version contains supplementary material available at 10.1186/s12906-025-04933-8.

## Clinical trial number

Not applicable.

## Introduction

Changes in the expression of glucose transporters and incretin secretion affect the intestinal absorption of glucose. Studying those changes has high biomedical relevance in the pathophysiology of type 2 diabetes and, potentially, other metabolic disorders. In mammals, intestinal glucose absorption occurs traditionally via SGLT1, which transports glucose from the intestinal lumen to the cytosol of enterocytes, whereas GLUT2 mediates glucose transports across the basolateral membrane into the interstitium and thereby into the circulation [1]. Besides their role in glucose transport, both sodium-glucose cotransporter 1 (SGLT1) and glucose transporter 2 (GLUT2) function as glucose sensors in entero-endocrine cells, leading to glucose-induced secretion of the incretin hormones glucose-dependent insulinotropic polypeptide (GIP) and glucagon-like peptide 1 (GLP-1) from intestinal L cells and K cells, respectively [2]. The pancreatic cells then respond to these hormones by increasing insulin release in a glucose-dependent manner. Preclinical studies found that these incretin hormones also regulate lipid metabolism, reduce appetite, and increase pancreas cell mass [3]. All these effects contribute to the anti-hyperglycemic effect of GLP-1 receptor agonists, an antidiabetic drug.

Vinegar is one of the most widely consumed functional foods for improving glycemic control throughout Asia [4]. Experimental trials in humans and some animal studies have demonstrated that the consumption of vinegar can improve postprandial glycemia and postprandial insulinemia [5, 6]. Recently, Cheng et al. [7] reported that vinegar intake can significantly improve glycemic control in patients with type 2 diabetes. *Nypa fruticans* Wurmb. vinegar, locally known as nipa palm vinegar (NPV), is one of the traditional vinegars produced by the fermentation of ‘nira’, nipa palm (*Nypa fruticans* Wurmb.) sap. The effects of aqueous extract (AE) of NPV on intestinal glucose absorption have been assessed through in vitro glucose absorption assays, in vivo carbohydrate tolerance tests, and enzyme inhibition studies. At a concentration of 1 mg/mL, AE demonstrated comparable results to phlorizin (1 mM), effectively delaying glucose absorption in isolated rat jejunum, outperforming acarbose (1 mg/mL). In vivo experiments showed that AE (500 mg/kg) significantly suppressed postprandial hyperglycemia 30 min after administering glucose (2 g/kg), sucrose (4 g/kg), and starch (3 g/kg) in normal rats, corroborating the in vitro findings. However, in enzymatic assays, AE displayed relatively weak inhibitory activity against both α-glucosidase and α-amylase when compared to acarbose. These results suggest that AE impedes intestinal glucose absorption through mechanisms other than inhibiting carbohydrate-hydrolyzing enzymes [8]. In another study, the twice-daily administration of NPV aqueous extract (1000 mg/kg B.W.) for 12 days significantly (*P* < 0.05) lowered blood glucose levels in streptozotocin (STZ)-induced type 1 diabetic rats, achieving a 56.6% reduction from initial values [9]. Further research examined AE’s mechanisms in managing blood glucose levels in type 1 diabetic rats. AE, at doses of 500 and 1000 mg/kg B.W. significantly reduced (*P* < 0.05) blood glucose, total cholesterol, and triglyceride levels while enhancing serum insulin levels. Interestingly, immunohistochemical analysis of the pancreas revealed no evidence of β-cell regeneration, despite the notable increase in insulin production. However, AE-treated groups exhibited restored hepatic histoarchitecture, suggesting a hepatoprotective effect against STZ-induced liver damage. Additional studies using RIN-5 F cell culture indicated that AE stimulated insulin release at basal glucose concentrations (1.1 mM) [10].

The studies, however, did not explore the antihyperglycemic effects of nipa palm vinegar AE in a type 2 diabetes model. The International Diabetes Federation Report for 2024–2030 estimates that 5.02 million (18.9%) of Malaysia’s adult population live with diabetes, with 99.33% having a diagnosis of type 2 diabetes mellitus (T2DM). Given the significant contribution of T2DM to all diagnosed diabetes cases, it is critical to conduct a study using this disease model. Hence, in the proposed study, we aim to investigate possible interactions between the AE and the machinery responsible for the glucose lowering effect by focusing on the intestinal glucose absorption. We used a combination of high-fat diet and streptozotocin (HFD-STZ) to induce T2DM in rat models. To achieve the study’s aim, we first analyzed the effect of Nipa palm vinegar on the glycemic parameters after being exposed to repeated doses of nipa palm vinegar. Then, the levels of incretin hormones were determined. To further understand the mechanism related to incretin hormones simulation, the concentration of DPP4 inhibitor was measured and the expressions of GLUT2 and SGLT1 were quantified using RT-qPCR.

## Materials & methods

### Preparation of nipa palm vinegar

 NPV was supplied by a local producer from Titi Bakong, Yan, Kedah, Malaysia (5°48′9.42″ N, 100°22′35.32″ E). Dr. Rahmad Zakaria, at the Herbarium Unit, School of Biological Sciences, Universiti Sains Malaysia, has authenticated several parts of the nipa palm. The voucher specimens have been deposited at the same unit with the voucher number of USM.Herbarium 11,541. NPV is a fermentation product of nipa sap called “nira.” It was extracted using the liquid-liquid extraction method. Ethyl acetate and distilled water were used sequentially in the extraction process of NPV. NPV (500 ml) was distilled to a final volume of 250 ml at 37 °C using a vacuum rotary evaporator. Concentrated NPV was first extracted with ethyl acetate in a ratio of 1:1 using a separatory funnel. The ethyl acetate layer (upper layer) was then separated. The residual ethyl acetate layer was collected and extracted three times with ethyl acetate. The ethyl acetate layers were mixed to form an ethyl acetate extract (EE). The residue (lower layer) is eventually considered the AE. Both collected extracts were concentrated at 40 ^o^C using a vacuum rotary evaporator and lyophilized using a freeze dryer. Finally, the extracts were stored in the freezer at -4 ^o^C until further use in the designated experiment. According to previous studies that compared the blood glucose-lowering effects of NPV, its EE), and its AE in a type 1 diabetes rat model, the aqueous extract had a stronger effect on blood glucose levels than NPV and EE (9). Therefore, the focus of this study was on AE.

### Animal handling and induction of diabetes

Male *Sprague-Dawley* rats (200–220 g) were supplied by the Animal Research Centre, Advanced Medical and Dental Institute, USM. The rats were acclimatized for seven days in a controlled room temperature of 23 ± 2 °C, 55 ± 10% humidity, and a 12-h dark-light cycle with free access to food and water. Then, rats were randomly divided into two groups: the normal control group (NC) and the diabetic control group (DC). The NC group received a regular diet. The diabetic rats’ group was fed a high-fat diet containing 68.5% normal diet, 10% sucrose, 20% fat, 1% cholesterol, and 0.5% cholic acid. After 8 weeks of dietary intervention, the diabetic rats were fasted for 24 h and then received STZ (30 mg/kg) via intraperitoneal (i.p.) injection. The rats in the NC group received an equivalent volume of vehicles and normal saline. One week after injection, fasting blood glucose (FBG) levels were determined using a glucometer (Accu-Chek^®^ Performa, Germany). Rats with FBG levels above 7 mmol/L were selected for the antihyperglycemic study.

### In vivo antihyperglycemic study

Thirty-six rats were divided into six groups of six rats each (*n* = 6) and treated once daily for 28 days, as follows:

Group 1: Normal control rats received 10 ml/kg B.W. of normal saline (NC).

Group 2: Diabetic control rats received 10 ml/kg B.W. of normal saline (DC).

Group 3: Diabetic rats received 250 mg/kg B.W. of AE (AE250).

Group 4: Diabetic rats received 500 mg/kg B.W. of AE (AE500).

Group 5: Diabetic rats received 1000 mg/kg B.W. of AE (AE1000).

Group 6: Diabetic rats received 60 mg/kg/B.W. of phlorizin (PHZ).

The dose of AE was selected based on a previous study investigating its antihyperglycemic effects in a type 1 diabetic rat model. In that study, compared to the DC group, AE at doses of 500 and 1000 mg/kg body weight significantly reduced FBG, total cholesterol, and triglyceride levels (*P* < 0.05) while also improving serum insulin levels [10].

Body weight and FBG levels were assessed each week. At the end of the study, the rats were anesthetized with an intraperitoneal injection of ketamine/xylazine (0.2 ml/kg B.W.). While under anesthesia, they were humanely euthanized through terminal cardiac puncture, allowing for blood collection for biochemical analysis. Approximately, 15–20 ml blood were collected. Blood serum was separated through centrifugation at 5,000 rpm for 10 min. The serum was stored at -20 °C for subsequent insulin, lipid profiles, liver enzymes and incretin hormones tests. The liver and pancreas were harvested for histology studies. Jejunum was extracted and kept in RNA Later (Sigma, USA) for RT-PCR.

### Serum biochemical analyses

Fasting serum insulin (FINS) concentration was measured using ELISA kits (Elabscience, China). To analyze the lipid profile (total serum cholesterol (TC), triglycerides (TG), high-density lipoprotein cholesterol (HDL-C), and low-density lipoprotein cholesterol (LDL-C) and liver enzymes (ALT and AST) levels, the automatic biochemical analyzer (Biolis 24i Premium, Japan, and Biosystems BA400, Biosystems S.A. Spain) was used. Incretin hormone levels, including GIP and GLP-1, and DPP-4 inhibitor were determined using ELISA kits (Elabscience, China).

### Insulin resistance and ß-cell function indices

Homeostasis model assessment-insulin resistance (HOMA-IR) and ß-cell function (HOMA- ß) was calculated to measure the insulin resistance and ß-cell function of the rats fed the experimental diets using the following formula:

$$\:\text{H}\text{O}\text{M}\text{A}-\text{I}\text{R}:\:[\text{F}\text{I}\text{N}\text{S}\:\times\:\:\text{F}\text{B}\text{G}]/22.5$$

$$\:\text{H}\text{O}\text{M}\text{A}-\:\ss:\:[20\:\times\:\:\text{F}\text{I}\text{N}\text{S}]/[\text{F}\text{B}\text{G}\:\--\:3.5]$$

To convert insulin from pg/L to pmol/L, a conversion factor 0.1722 was employed (provided in the user’s manual of the ab278123 Human Insulin SimpleStep ELISA^®^ Kit). Additionally, the relationship 1µU/mL equal to 6.00 pmol/L was used to convert from pmol/L to mIU/L [11].

### H&E staining for liver and pancreas tissues

Liver and pancreatic tissues were fixed in 10% formalin, embedded in paraffin wax, and cut into 5 μm-thick sections. The sections were then put on slides and deparaffinized with xylene I and xylene II for two minutes each. After that, the slides were dehydrated with a decreasing percentage of alcohol (100% alcohol, 95%, 80%, 70%, and 50%) and put in running tap water for two minutes. To stain the cell nuclei, the slides were immersed in Harris Hematoxylin for 15 min, followed by a one-minute immersion in running tap water. Differentiation of tissues was done by dipping in 0.5% acid alcohol (2 dips) and 1 min of running tap water. The process of tissue blueing involved immersing the slides in 0.3% ammonia for one minute, then immersing them in running tap water for another minute, and finally dipping them ten times in 95% alcohol. Counterstained were done by immersing in eosin (Bendenson, Malaysia) for ten minutes, and the tissues were dehydrated in increasing grades of alcohol (95% alcohol I, 95% alcohol II, 95% alcohol III, 100% alcohol I, and 100% alcohol II). Slides were cleared by dipping them three times in xylene I and xylene II and mounted with DPX. The sections of liver tissue were examined under a light microscope (HumaScope Classic, Germany) with a digital camera attached. Digital photomicrographs were taken at 400x magnifications.

Stained sections of pancreas were analyzed using inverted microscope (Olympus IX51, Japan) with a digital camera attached and the photomicrographs of pancreatic islets of Langerhans were taken using Charge-Coupled Device (CCD) camera at 400× magnifications. The measurement of the diameter of pancreatic islets was carried out on photomicrographs by calculating the mean of islets diameter for each section, and then the mean of diameters for many sections and finally the mean for all animals within the same group using the Toupview camera Software.

### Gene expression of intestinal glucose transporters

#### RNA extraction and cDNA synthesis

Jejunal samples were stored in RNAlaterTM (Sigma, USA) at − 80 °C until use. For total RNA extraction from tissue samples, 30 mg of samples (stored at − 80 °C) were thawed on ice and crushed with a mortar and pestle under liquid nitrogen. The powders of tissue were obtained, and total RNA was extracted using the NucleoSpin^®^ RNA Extraction Kit (Macherey Nagel, Germany) according to the manufacturer’s instructions. First-strand complementary DNA (cDNA) conversion was conducted using the OneScipt Hot Reverse Transcriptase Kit (ABM Good, Canada). Briefly, 1 ng of total RNA was mixed with 4 µL of 5x RT buffer, 1 µL of dNTP, 1 µL of OneScript Hot RTase, and 1 µL of random primer. The mixture was mixed and centrifuged for 30 s under 10,000 rpm. The mixture is then incubated for 15 min at 60 °C, followed by 5 min at 85 °C. The synthesized cDNA was stored at -20 °C.

#### Gene expression analysis: real time quantitative PCR (RT- qPCR)

Analysis of the samples was conducted using qPCR Mastermix (BlasTaq™ 2X qPCR MasterMix, Canada) protocols. The primer was obtained from Integrated DNA Technologies, USA. The primer pair was 5′-TAC CTG AGG AAG CGG TTT GGA-3′ (forward) and 5′-CGA GAA GAT GTC TGC CGA GA-3′ (reverse) for SGLT1, and 5′-GAG TTC CTT CCA GTT CGG CTA TG-3′ (forward) and 5′-GTT CCA CTG GAT GAC CGG-3′ (reverse) for GLUT2. The amount of GLUT2, and SGLT1 mRNA was normalized to the amount of mRNA of the housekeeping gene, GADPH. The primer pair for GADPH was: 5′-TGC TGG TGC TGA GTA TGT CG-3′ (forward) and 5′-TTG AGA GCA ATG CCA GCC-3′ (reverse). Following the directions on the package, 6.5 µl of nuclease-free water, 10 µl of PCR Master Mix (2x), 0.5 µl of forward and reverse primers each containing 0.5 µM of DNA, and 10 ng/µl of cDNA template were used in the qPCR reaction. The endpoint was conducted as follows: Enzyme activation: 95 °C at 30 min in one cycle; denaturation: 95 °C at 50 s in 40 cycles; annealing: 60 °C at 1 min; melting curve: 60–95 °C in rate: 0.2 °C/s.

### Statistical analysis

The data were presented as the mean ± SEM. The statistical analysis was performed using SPSS. Continuous variables with a parametric distribution were analyzed using Analysis of Variance (one-way ANOVA), and if the results were significant, a post-hoc Dunnett’s test was performed. A Kruskal-Wallis’s test and a post-hoc Mann-Whitney U test were conducted for data with non-parametric distributions. A P-value less than 0.05 was considered statistically significant.

## Results

### Effects of AE on body weight

As compared to their initial value, the NC group demonstrated a significant increase in body weight (8.86%) by the end of the 4-week treatment period. In contrast, diabetic conditions resulted in weight loss with DC rats showing a 3.13% decrease from their initial weight. A similar trend was observed in diabetic rats treated with AE and PHZ. Compared to the NC group, DC rats exhibited a significant (*p* < 0.05) 20.99% reduction in body weight. Likewise, PHZ- and AE-treated diabetic rats showed a significant reduction in body weight, except for the AE500-treated group. Furthermore, compared to DC group, only NC exerted significant body weight increase. After 28 days of treatment, no significant changes in body weight were observed in any of the PHZ- or AE-treated groups. The data of body weight changes are presented in Table 1.

**Table 1:** Body weight changes of all experimental groups, before and after 28 days of intervention

Group	Body weight (g)	
	Initial	Final
NC	539.67±22.76	587.50±23.60*^$^
DC	479.00±20.62	464.17±16.01^#^
PHZ	468.00±16.01	456.50±39.07*^#^
AE250	475.50±23.75	460.50±39.14^**#**^
AE500	483.17±17.31	464.50±31.50^#^
AE1000	463.67±14.40	431.50±25.15*

Groups consisted of normal control (NC), diabetic control (DC), phlorizin 60 mg/kg B.W. (PHZ), and AE at the doses of 250 mg/kg B.W. (AE250), 500 mg/kg B.W. (AE500), and 1000 mg/kg B.W. (AE1000). Data were expressed as mean ± SEM (*n* = 6). All the data were statistically significant at *p* < 0.05. *P < 0.05 compared to the initial value using paired t-test. ^$^*P* < 0.05 compared to DC and ^#^*P* < 0.05 compared to NC using Dunnett post-hoc test

### Effect of AE on fasting blood glucose and serum insulin levels

Administration of HFD/STZ increased the fasting blood glucose of rats. The average blood glucose levels of the NC group were 5.42 mmol/L, while the DC group had significantly higher average FBG of approximately 19.48 mmol/L. Over the 28 days of treatment period, the FBG reading in NC decreased, from the initial value of 5.42 to 5.02 mmol/L. The decrease, however, was not significant. As expected, DC showed elevated FBG, and the value remained high throughout the period. AE treatment, at the dose of 1000 mg/kg B.W. caused a significant decrease (*p* = 0.015) in FBG, as compared with the initial value, with the highest percentage decrease of 34.26% (Table 2). As for serum insulin level, NC rats exerted the highest concentration of 358.06 ± 9.47 pg/ml, while DC rats showed a decrease in insulin levels (167.55 ± 24.14 pg/mL). Compared to DC, the administration of AE at the doses of 250 and 1000 mg/kg resulted in a significant elevation of serum insulin level (*p* = 0.003 and *p* = 0.001, respectively).

**Table 2:** Effects of AE on fasting blood glucose (FBG) and serum insulin of T2DM after 28 days of treatment

Group	FBG after STZ induction (mmol/L)	FBG after 28 days (mmol/L)	Mean decrease in FBG	Percentage decrease in FBG (%)	Insulin Concentration (pg/mL)
NC	5.42 ± 0.21^**#**^	5.02 ± 0.15^**#**^	0.4 ± 0.24	6.58 ± 4.93	358.06 ± 9.47^#^
DC	19.48 ± 4.94*	20.05 ± 3.33*	-0.57 ± 2.98	-26.95 ± 25.56	167.55 ± 24.14*
PHZ	17.15 ± 2.17*	12.28 ± 2.95	4.87 ± 1.23	32.64 ± 10.25	287.81 ± 18.68^#^
AE250	13.84 ± 2.06	11.63 ± 2.76	4.15 ± 2.51	21.12 ± 21.39	310.66 ± 25.84^#^
AE500	19.92 ± 2.12*	14.48 ± 3.03	5.44 ± 3.60*	17.77 ± 26.19	258.85 ± 37.00*
AE1000	20.85 ± 3.63*	11.38 ± 1.78^**#**^	9.47 ± 4.15*	34.26 ± 16.56^**#**^	330.32 ± 19.59^#^

NC represents normal control rats, while PHZ refers to the group treated with 60 mg/kg B.W. phlorizin. AE250, AE500, and AE1000 indicate rats treated with AE at doses of 250 mg/kg B.W., 500 mg/kg B.W., and 1000 mg/kg B.W., respectively. Data are expressed as mean ± SEM (*n* = 6). *P < 0.05 compared to NC; #*P* < 0.05 compared to DC, as analyzed using one-way ANOVA with Dunnett-Tukey’s post hoc test

### Effects of AE HOMA-IR and HOMA- ß cell function indices

**Fig. 1:**
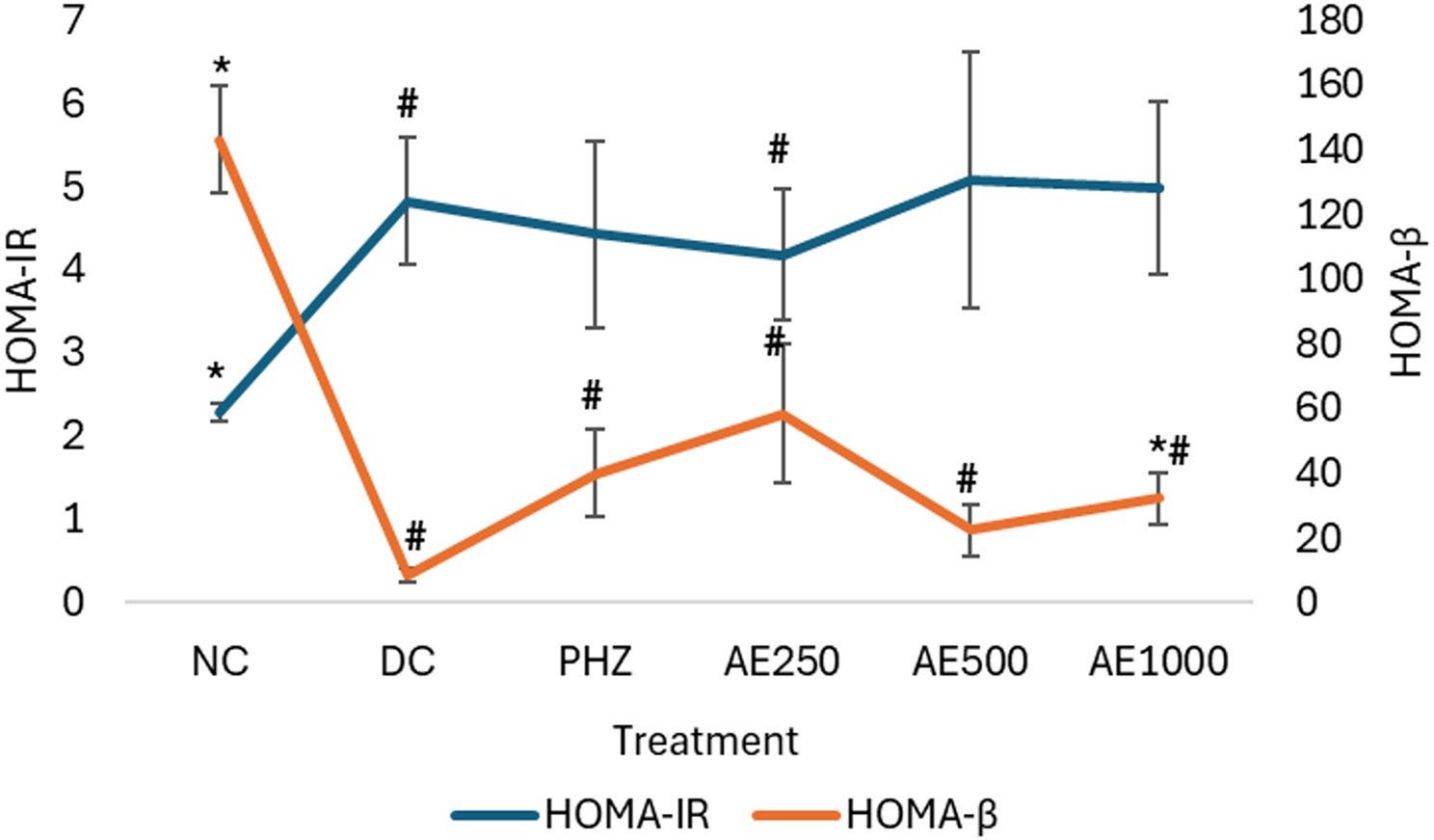
Effects of AE on HOMA- ß and HOMA-IR index after 28 days of treatment with AE. Data were expressed as mean ± SEM (*n* = 6). Groups consisted of normal control (NC), diabetic control (DC), phlorizin, 60 mg/kg B.W. (PHZ), and AE at the dose of 250 mg/kg B.W. (AE250), 500 mg/kg B.W. (AE500), and 1000 mg/kg B.W. (AE1000). All the data were statistically significant at #*p* < 0.05 vs. NC and *p < 0.05 vs. DC, analyzed using non-parametric Kruskal Wallis with Mann Whitney-U post hoc test

The HOMA-IR index indicated the presence of insulin resistance in DC as the score was significantly (*p* = 0.004) increased in the group compared to that of NC group. No statistical difference was found in the HOMA-IR index among the DC and AE-treated groups. However, HOMA- ß cell function of DC was significantly reduced, as compared to NC group. AE at the dose of AE1000 significantly improved the ß cell function when compared to DC with the respective p values of 0.004 (Fig. 1).

### Effects of AE on dyslipidemia

As shown in Table 3, diabetes led to significant elevation in serum levels of CHOL, TG, HDL, and LDL when compared to the NC group. Treatment with AE significantly normalized the lipid profile of diabetic-treated rats, as levels of CHOL, TG, and LDL were significantly reduced (*p* < 0.05) when compared to DC group. This indicated that the treatment had a positive effect on restoring the lipid levels. Similar effects were observed in the PHZ-treated group. The HDL level of all diabetics treated remained unchanged.

**Table 3:** Lipid profiles of type 2 diabetic rats after being treated with Nipa palm vinegar for 28 days. The group consisted of normal control (NC), diabetic control (DC), 60 mg/kg B.W. Phlorizin (PHZ), and Nipa palm vinegar with doses of 250 mg/kg B.W. (AE250), 500 mg/kg B.W. (AE500), and 1000 mg/kg B.W. (AE1000)

Total concentration (mmol/L)				
Group	CHOL	TG	HDL	LDL
NC	1.23 ± 0.13**	0.51 ± 0.07**	0.38 ± 0.06*	0.23 ± 0.02**
DC	10.20 ± 6.16^##^	2.55 ± 0.90^##^	0.63 ± 0.22^#^	4.41 ± 3.20^##^
PHZ	2.39 ± 0.56**	0.68 ± 0.06*	0.57 ± 0.04	0.52 ± 0.15**
AE250	2.26 ± 0.03**	0.53 ± 0.06*	0.42 ± 0.02	0.49 ± 0.03**
AE500	2.39 ± 0.32*^#^	0.75 ± 0.08*	0.57 ± 0.07	0.53 ± 0.08*^#^
AE1000	2.20 ± 0.26**	0.86 ± 0.33*	0.56 ± 0.10	0.40 ± 0.03*

Data are presented as mean ± SEM (*n* = 6). Statistical significance is indicated as *P < 0.05, **P < 0.01 compared to DC and ^#^*P* < 0.05, ^##^*P* < 0.01 compared to NC. Based on data normality, HDL was analyzed using one-way ANOVA with Dunnett’s post hoc test, while TG, CHOL, and LDL were analyzed using the Kruskal-Wallis test with Mann-Whitney U post hoc test. CHOL: Cholesterol; TG: Triglycerides; HDL: High-density lipoprotein; LDL: Low-density lipoprotein

### Effects of AE on liver histopathology and serum liver enzymes

**Fig. 2:**
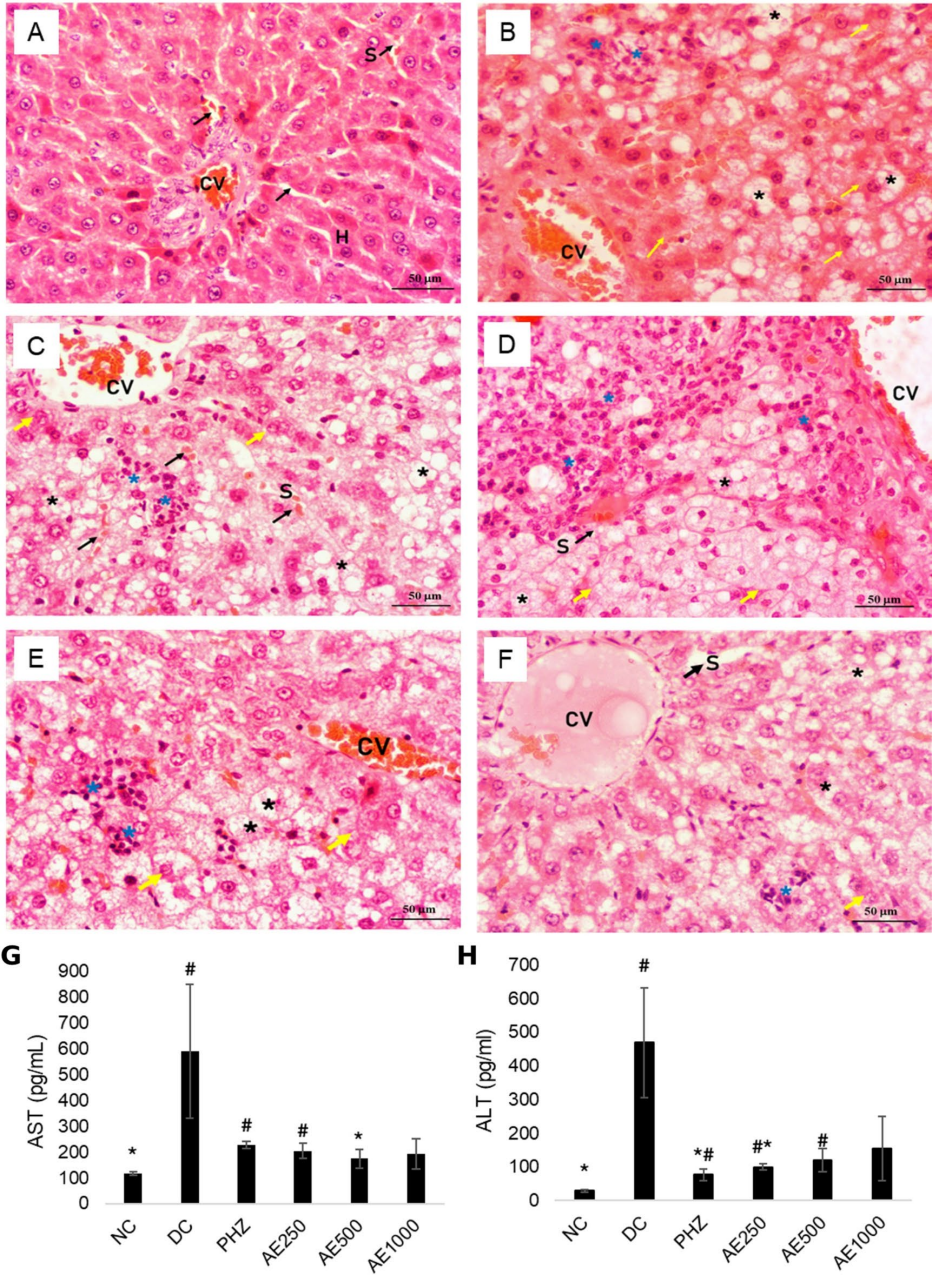
Effects of high-fat diet and treatment of AE on histopathological changes in liver tissues (stained with hematoxylin and eosin dye). (**A**) Normal control (NC): Rats fed with normal diet; (**B**) Diabetic control (DC): Rats given HFD-STZ; (**C**) 60 mg/kg B.W. (PHZ): Rat given HFD-STZ; (**D**)(**E**)(**F**) Rat given HFD-STZ and treated with the AE at the respective dose of 250 mg/kg B.W. (AE250), 500 mg/kg B.W. (AE500), and 1000 mg/kg B.W. (AE1000), (**G**) Concentration of serum alanine transaminase (ALT) and (**H**) aspartate transaminase (AST). Images were observed under 400x magnification. Pleomorphic and multinucleated (H); Central hepatic veins (CV); Sinusoids (S); Hepatocytes (black arrow); Ballooning (yellow arrow); Steatosis (*); Lobular inflammation (*)

Figure 2 illustrates AE’s effects on liver histopathology and liver enzyme levels. The NC group had polygonal hepatocyte cells with round, centrally located nuclei, as well as abundant eosinophilic cytoplasm with fine basophilic granules. Central hepatic veins were lined by endothelium, whereas sinusoids were lined by endothelial cells, Kupffer cells, and separate cords of hepatocytes (Fig. 2A). DC showed enlarged and balloning hepatocytes with pale cytoplasm, glycogenated nuclei, and accentuated cytoplasmic membranes. Steatosis was abundant, and lobular inflammation may be present (Fig. 2B). The PHZ, AE250, and AE500-treated groups exhibited similar features, including enlarged or ballooning hepatocytes with pale cytoplasm, glycogenated nuclei, and accentuated cytoplasmic membranes. However, steatosis was not as severe as in the DC group, and lobular inflammation may be present. The sinusoid was still intact, partially in the sheet-like appearance of the hepatocyte architecture. The AE1000-treated group experienced a reduction in hepatocyte ballooning. Steatosis decreased to almost normal, and lobular inflammation was scarce. Sinusoids lined with endothelial cells and Kupffer cells may be present. Figure 2 (G) (H) indicated that the liver markers of AST and ALT in DC rats were significantly higher (*p* = 0.05) than in NC rats. AE had decreased AST and ALT levels, with a significant reduction seen in the respective AE-500 and AE-250 groups as compared to DC.

### Effects of AE on pancreatic islet

**Fig. 3:**
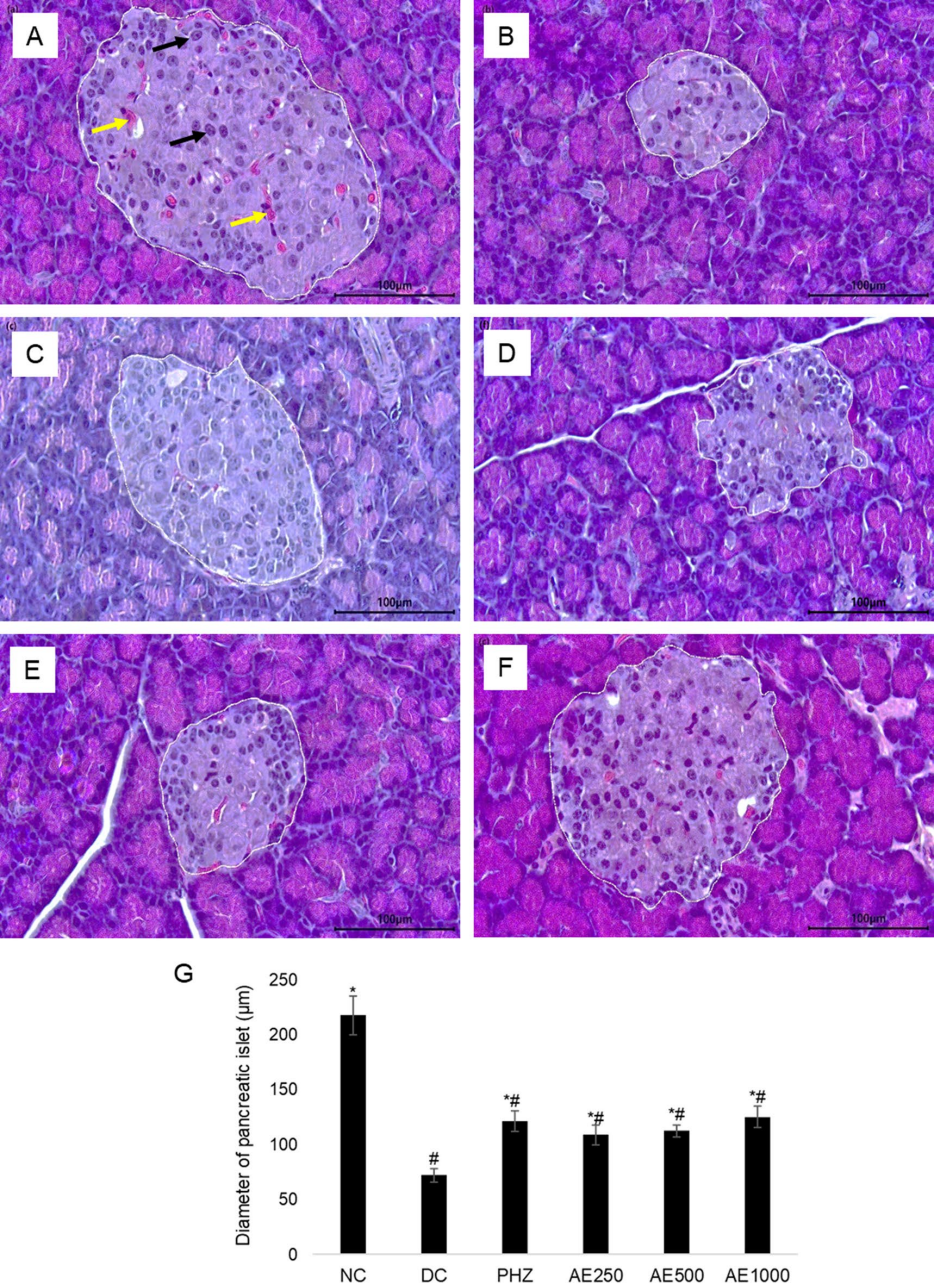
Photomicrographs of pancreatic islets of Langerhans and average diameter islets at 400x magnification after 28 days of daily oral treatment. The area of the islet was outlined in white. (**A**) NC: Normal control; (**B**) DC: Diabetic control; (**C**) PHZ: 60 mg/kg B.W. Phlorizin; (**D**) AE250: 250 mg/kg B.W. of AE; (**E**) AE500: 500 mg/kg B.W. of AE; (**F**) AE1000: 1000 mg/kg B.W. of AE; (**G**) Mean of islet diameter. Pancreatic islet cells (black arrow); Capillaries (yellow arrow). Values were expressed as means ± SEM, *P < 0.05 compared to DC; #*P* < 0.05 compared to NC, analysed using Kruskal Wallis with Mann Whitney U as post hoc test

Histological sections of the pancreas and average islet diameter are presented in Fig. 3. Photomicrographs of the pancreas from NC group showed normal architecture with normal acini and a normal population of the islets of Langerhans cells (A). Pancreatic islet of DC became hypertrophic with a reduced number of islet cells and irregular appearance of the islet (B). The results obtained showed that administration of AE attenuated the destructive effect of HFD-STZ on pancreatic islets and induced a restoration of the normal cellular population of islet cells (D, E, F). Group treated with phlorizin showed less restoration of cells of islets of Langerhans and partial regeneration of islets cells (C). The results of morphometric analysis of pancreas showed that HFD-STZ administration induced a significant reduction in the islet diameter (72.09 ± 6.38 μm), compared to NC group (217.86 ± 17.61 μm). Whereas the oral administration of AE for 28 days prevented significantly the HFD-STZ induced pancreatic islet damages by preserving islet diameter of 108.85 ± 8.79 μm, 112.56 ± 5.42 μm, and 125.27 ± 9.52 μm in the respective group of AE250, AE500 and AE1000.

### Effects of AE on incretin hormones and DPP4 inhibitor

**Fig. 4:**
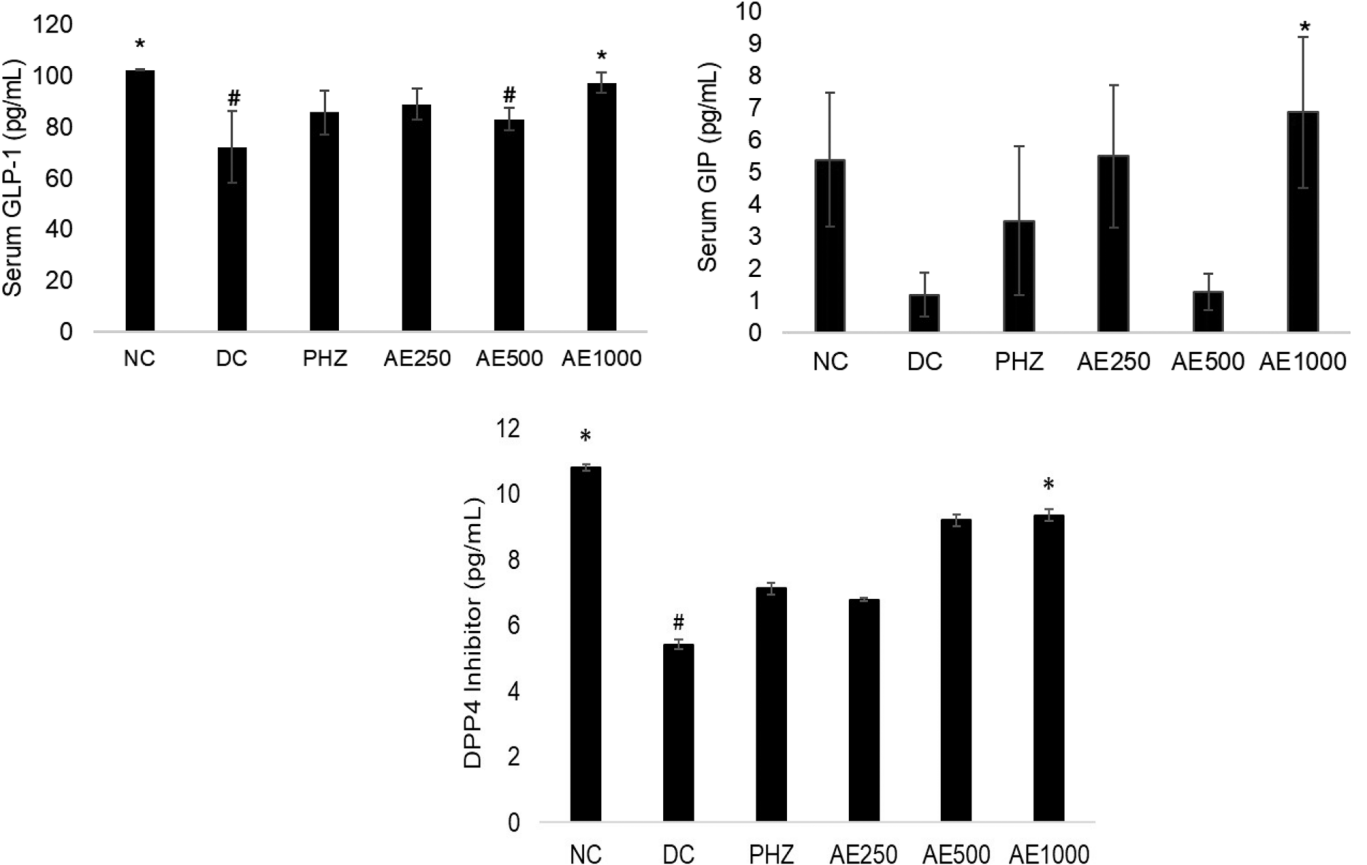
Effects of nipa palm vinegar treatment on serum levels of GLP-1, GIP and DPP4 inhibitor. The group comprised normal control (NC), diabetic control (DC), 60 mg/kg B.W. phlorizin (PHZ), and nipa palm vinegar with doses of 250 mg/kg B.W. (AE250), 500 mg/kg B.W. (AE500), and 1000 mg/kg B.W. (AE1000). Data are represented as means ± SEM (*n* = 3). The data were statistically significant at ^#^*P* < 0.05 compared to NC and *P < 0.05 compared to DC, analysed using one-way ANOVA with Dunnett as post hoc test

Figure 4 illustrates the effects of AE treatments on GIP, GLP-1, and DPP4 inhibitor. DC recorded the lowest concentration of GIP, GLP-1 and DPP4 inhibitor. At a dose of 1000 mg/kg, AE significantly improved the levels of GIP, GLP-1 and DPP4 inhibitor, as compared to the DC.

### Gene expression of SGLT1 and GLUT2

**Fig. 5:**
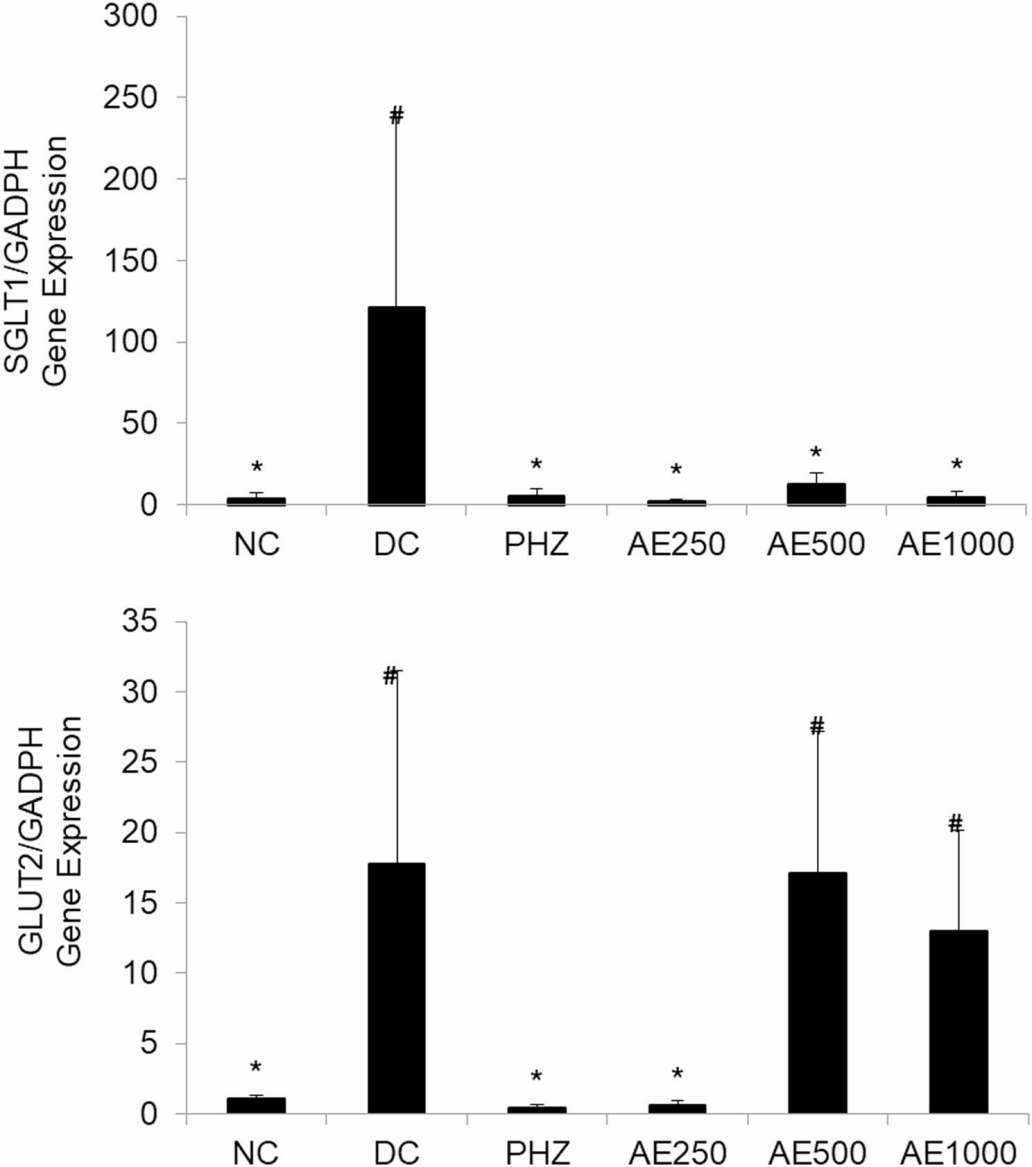
Real-time qPCR of the mRNA expression levels of SGLT1 and GLUT2 after AE treatment. Groups consist of normal control (NC), diabetic control (DC), 60 mg/kg phlorizin (PHZ), and AE-treated groups of 250, 500, and 1000 mg/kg B.W. (AE250, AE500, AE1000). GAPDH was used as a housekeeping gene. The data were statistically significant at *P < 0.05 compared to DC; #*P* < 0.05 compared to NC, analysed using Kruskal Wallis with Mann Whitney U as post hoc test

Figure 5 shows the effect of AE on intestinal glucose transporters, SGLT1, and GLUT2. Diabetes induction led to the overexpression of SGLT1, and GLUT2 (*p* = 0.03, *p* = 0.051, respectively). As compared to DC, treatment with AE at the dose of 500 and 1000 mg/kg B.W. significantly downregulated the expression of SGLT1. On contrary, AE at the similar doses did not affect the expression of GLUT2. PHZ, known as SGLT1/2 inhibitor, downregulated the expression of both genes.

## Discussion

The aim of this study was to characterize possible interactions between the AE of nipa palm vinegar and the machinery responsible for the intestinal glucose absorption using a high-fat diet and streptozotocin (HFD-STZ)-induced T2DM rat. Research suggested that administering HFD for a specific duration triggered insulin resistance, while a low dose of STZ induces mild impairment in insulin secretion. Thus, a combination of HFD and a low dose of STZ establish a T2DM model that closely replicates the metabolic characteristics seen in people with T2DM [12]. The successful establishment of such a model would be cheaper compared to genetically modified models and practical for laboratory testing.

The present study induced T2DM in rats by feeding them a HFD for 8 weeks, followed by an intraperitoneal injection of STZ at a low dose of 30 mg/kg body weight. The success of the T2DM model induction was confirmed by the presence of key metabolic abnormalities in DC, including hyperglycemia (FBG > 7 mmol/L), insulin resistance (indicated by an elevated HOMA-IR index of 4.82), and disrupted lipid profiles compared to the NC group. These findings aligned with those reported by Assadi et al. [13], who utilized a similar diabetic model. Those abnormalities were ameliorated after 28 days of treatment with AE. AE at a dose of 1000 mg/kg had the best efficacy in controlling hyperglycemic effects. Though treatment with the AE did not return the blood glucose level to normal, the level of reduction was statistically significant when compared to before treatment with AE. A previous study on the antidiabetic effects of AE in the type 1 diabetes mellitus model supported this finding, showing that 1000 mg/kg of AE was the most effective in lowering blood glucose levels [9].

To further investigate the glucose-lowering mechanism of AE, we assessed several key parameters, including serum insulin concentration, HOMA-β, HOMA-IR, histology of pancreatic islets and hepatocytes, incretin hormones, DPP4 inhibitor, and the expression of glucose transporters, GLUT2 and SGLT1. The results demonstrated that AE effectively restored insulin levels in diabetic rats. Specifically, the AE1000-treated group exhibited significantly higher insulin levels compared to the DC group. This improvement in insulin levels suggests a potential recovery of β-cell function, as evidenced by improvement in the HOMA-β index, and pancreatic islet morphology. These findings suggest that AE protects β-cell from further damage induced by STZ and facilitate the functional recovery of β-cells, thus restore insulin secretion in diabetic rats. As a well-established diabetogenic agent, STZ induces pancreatic β-cell destruction and dysfunction by selectively targeting pancreatic β-cells through the GLUT2 glucose transporter. It induces DNA alkylation and oxidative stress by generating reactive oxygen and nitrogen species. These mechanisms lead to DNA damage in pancreatic β-cells, ultimately resulting in cell death [14]. In our study, we observed that STZ injection resulted in significant β-cell degeneration, as evidenced by reduced insulin levels, lowered HOMA-β index and histopathological changes in pancreatic islet of the DC group. Similar findings have been observed in studies that applied HFD-STZ-induced type 2 diabetes rat models [15, 16]. AE treatment, however, mitigated these effects by preserving β-cell mass and function, as demonstrated by improved insulin levels, HOMA-β and pancreatic morphology. Notably, the average size of pancreatic islets in the AE-treated group was significantly larger, suggesting cellular restoration within the Langerhans islets. The β-cell protective effects of AE may be attributed to its antioxidant and anti-inflammatory properties. Oxidative stress and inflammation are major contributors to β-cell damage in T2DM [17]. Several bioactive compounds of nipa palm vinegar, namely gallic acid, protocatechuic acid, and 4-hydroxybenzoic acid, have been shown to possess antioxidant and anti-inflammatory effects that could counteract these detrimental processes [18]. By reducing oxidative stress and inflammation, these bioactive compounds may help restoring β-cell function, thereby enhancing insulin secretion. Several studies have demonstrated the beneficial effects of functional foods in preserving pancreatic β-cell function. For example, Gallardo-Villanueva and colleagues [19] reported that flavonoid-rich cocoa-carob flour supplementation prevented β-cell apoptosis and the loss of beta cells in Zucker diabetic fatty rats by counteracting pancreatic oxidative stress and suppressed islet inflammation. Similarly, Tsai et al. [20] reported that the powdered root of *Eurycoma longifolia* Jack enhanced β-cell proliferation and improved pancreatic islet function through the induction of PDX1, thereby contributing to the plant’s antihyperglycemic properties.

Restoring impaired insulin secretion is a crucial factor in improving glycemic control in patients with type 2 diabetes mellitus. Insulin plays a pivotal role in regulating glucose homeostasis by suppressing hepatic glucose production through the inhibition of gluconeogenesis and glycogenolysis. Additionally, insulin promotes glucose uptake in muscle and adipose tissues, thereby reducing blood glucose levels and ameliorating hyperglycemia [21]. Therefore, the upregulation of insulin secretion may represent a key mechanism through which AE exerts its antidiabetic effects in T2DM. As fasting blood glucose and insulin levels improved, HOMA-IR index in AE-treated groups, however, remained high and was not significantly different from DC group, further suggesting persistent insulin resistance. These findings imply that AE may be acting selectively on pancreatic β-cells without affecting key insulin resistance pathways namely AMPK, PI3K/Akt, or GLUT4 translocation in peripheral tissues including liver, muscle and adipose. Additionally, the findings also suggested that the duration of the plant extract treatment might be insufficient to induce changes in peripheral insulin sensitivity, which often requires longer-term interventions to reverse metabolic dysfunction. Several plant species have been reported to improve insulin resistance in diabetic models after prolonged treatment, including *Litchi chinensis Sonn.* (6 weeks of treatment), *Momordica charantia* L. (8 weeks of treatment), *Phellinus linteus* (8 weeks of treatment), and *Zingiber officinale* (8 months of treatment) [22]. Additionally, a review of clinical trials conducted by Li et al. [23] on the therapeutic mechanisms of herbal medicines against insulin resistance found that 66% of studies reported significant improvements in insulin resistance after 12 weeks of treatment. These discrepancies highlight the critical influence of treatment duration on study outcomes.

HFD-STZ induced dyslipidemia in the rats as indicated by an imbalance of blood lipids, including high levels of low-density lipoprotein cholesterol (LDL-C) and/or triglycerides (TG), and/or low levels of high-density lipoprotein cholesterol (HDL-C) [24]. Restoration of insulin concentration may correct those abnormalities in lipid metabolisms in diabetic rats, further explaining how AE managed to normalize dyslipidemia in diabetic rats. Dyslipidemia in diabetes is common irrespective of insulin deficiency or insulin resistance [25]. Insulin deficiency or resistance activates intracellular hormone-sensitive lipase, an enzyme that breaks down triglycerides stored in metabolically active central adipose tissue, releasing non-esterified fatty acids (NEFA) into the bloodstream. Elevated NEFA levels stimulate the liver to produce more triglycerides, leading to an increased secretion of apolipoprotein B (apoB), a key component of very-low-density lipoprotein (VLDL) [26]. Furthermore, the inhibitory effect of insulin on hepatic apoB production and triglyceride secretion is absent, leading to enhanced VLDL secretion. The enzyme lipoprotein lipase, found on the vascular endothelium, plays a crucial role in removing triglycerides from circulation. In insulin resistance or deficiency, its activity may be downregulated, leading to slower triglyceride clearance and contributing to postprandial lipemia [27]. Insulin is essential for regulating lipid metabolism and mitigating dyslipidemia in diabetic rats. Insulin inhibits excessive lipolysis in adipose tissue, thereby lowering NEFA that contribute to hepatic triglyceride synthesis and VLDL overproduction [25].

Improvements in glucose and lipid metabolism were reflected in the hepatocytes as the liver plays a vital role in glucose and lipid metabolism by regulating the uptake, synthesis, and release of glucose and lipoproteins into the system [28]. A high-fat diet combined with STZ can induce liver injury and changes in hepatocytes, leading to alterations in liver function and morphology, including increased liver enzymes and potential for non-alcoholic fatty liver disease (NAFLD) [29]. NAFLD activity score showed the presence of steatosis, lobular inflammation and ballooning in the liver tissue of DC group (Supplementary file). Histological images of the liver tissue of diabetic rats treated with AE1000 showed less hepatocyte ballooning and steatosis (lipidosis and fatty changes), and there were fewer signs of lobular inflammation than in untreated DC. The improved morphology features indicated that treatment with AE reduced hepatic lipid accumulation and improved hepatic insulin resistance of diabetic rats. AE also exerted a hepatoprotective effect by restoring the level of liver enzymes, ALT, and AST. As increase in liver enzymes indicating hepatocellular injury associated with insulin resistance, metabolic syndrome, and T2DM [30, 31], Restoration of liver enzyme levels following AE treatment indicates its hepatoprotective effects.

Incretin hormones are known to exhibit insulinotropic and non-insulinotropic effects. Hence, the levels of these hormones were examined in the present work to further understand how AE stimulated insulin secretion and preserved pancreatic β-cells. GLP-1 and GIP are gut peptides secreted by L cells (GLP-1) and K cells (GIP); and they are primarily triggered by the presence of carbohydrates (glucose), proteins, triglycerides, and fatty acids in the gut [32]. The insulinotropic properties of GLP-1 and GIP are conferred through potentiating GIPR and GLP-1R, cell-membrane G-protein-coupled receptors, in β-cells. When these receptors are activated, they dissociate the α-subunits of G-proteins, resulting in a transactivation by adenylate cyclase. Consequently, ATP dephosphorylation ensues yielding higher levels of cyclic AMP, which in turn trigger protein kinase A. These protein subunits close the K^+^ ion-gated channels in the membrane, resulting in the influx of Ca^2+^ ions through voltage-gated Ca^2+^ channels, depolarizing β-cells’ membranes, which triggers insulin secretion by those β-cells [33]. The non-insulinotropic effects of GIP and GLP-1, on the other hand, have been observed to include the inhibition of necro-apoptosis of β cells and the proliferation and neogenesis of pancreatic β cells in INS-1 cells in vitro and in vivo, in mice and in Vancouver diabetic fatty Zucker rat models [34]. In the diabetic state, the levels of incretin hormones are markedly diminished [35]. These observations were in line with the effect observed in the DC of this study. Our AE treatment, particularly at a 1000 mg/kg B.W. dose, normalized the levels of GLP-1 and GIP. Based on these findings, it can be hypothesized that insulin stimulation and the pancreatic-protective effects of AE could be due to an enhanced release of incretin hormones.

In our study, several mechanisms may contribute to incretin hormone release. A key mechanism involves the inhibition of dipeptidyl peptidase-4 (DPP-4) by DPP-4 inhibitors. Endogenous GLP-1 and GIP are rapidly degraded by DPP-4 by removing a dipeptide from their N-termini, limiting their plasma half-lives to approximately 7 min and 1–2 min, respectively [36]. This degradation reduces their insulinotropic function. Inhibiting DPP-4 extends the half-life of incretin hormones, enhancing pancreatic islet response and improving glucose homeostasis. Therefore, DPP-4 inhibitors are considered an important therapeutic target for T2DM. Our results demonstrated that the AE-treated groups exhibited significantly higher levels of DPP-4 inhibitors compared to the DC group. This increase corresponds with the elevated incretin hormone levels observed in these groups. Notably, nipa palm vinegar contains several bioactive compounds, including gallic acid, rutin, catechin, and quercetin [37]. These compounds have been reported to exert significant DPP-4 inhibitory activity by competitively or non-competitively binds to the DPP4 enzyme’s active site and interacting with key residues in the S1, S2, and S3 sites through hydrogen bonding, ionic or polar interactions, and Van der Waals forces. These interactions induce conformational changes in DPP-4 or alter the side chains of its amino acid residues, lead to reduce enzyme’s catalytic activity when the substrate is also bound [38]. Besides preserving incretin hormones, several animal studies have shown that DPP4 inhibitor promoted islet neogenesis, β-cell regeneration and β-cell proliferation [39]. Accordingly, the AE-treated group showed a significant increase in serum insulin levels and pancreatic islet diameter, which suggested the restoration of islet cell number and function. This effect may be attributed to DPP4 inhibitors.

Furthermore, the data available suggest that SGLT1 and GLUT2 play a crucial role in glucose-dependent stimulation of GLP-1 and GIP secretion in the small intestine [40, 41]. Thus, we studied the expression of intestinal SGLT1 and GLUT2 to determine how AE affected these intestinal glucose transporters, which may help to explain the observed stimulation effect of AE on incretin hormones. In this study, DC upregulated the expression of SGLT1, which aligns with the findings of Zhu et al. [42], who observed increased SGLT1 mRNA expression in the duodenal and jejunal regions of Goto-Kakizaki diabetic rats. Treatment with AE at a dose significantly decreased SGLT1 expression, as compared to the DC. Conversely, AE at the dose of 500 and 1000 mg/kg did not cause any significant change on GLUT2 expression. The role of GLUT2 in stimulating incretin hormones remains inconclusive. Several studies have shown the importance of GLUT2 in stimulating GLP-1 and/or GIP secretion at very high glucose concentrations in the intestinal lumen [41, 43]. However, a study by Roder et al. [40] found no significant differences in GIP and GLP-1 secretion in GLUT2 knockout mice following an oral glucose gavage. This suggests that GLUT2 plays a limited role in K and L cell-mediated incretin secretion. Our findings aligned with those of Roder et al. [40], as the downregulation of GLUT2 by AE appeared to have minimal or no effect on incretin hormone secretion. Overall, the inhibition of SGLT1 contributes to the sustain release of incretin by AE. In the small intestine, SGLT1 mediates glucose and galactose absorption across the apical cell membrane. SGLT1 in L cells in proximal intestine sense dietary glucose, which subsequently triggers the acute release of GLP-1. SGLT1 inhibition in proximal intestine reduces glucose absorption. By limiting glucose absorption in the proximal intestine, glucose delivery to the distal gut increases, where it is metabolized by gut microbiota into short-chain fatty acids (SCFAs). These SCFAs activate free fatty acid receptors (FFAR2 and FFAR3) on distal L-cells, triggering a sustained release of GLP-1 [44].

This study has several limitations. The present study employed a 28-day treatment period to evaluate the effects of AE on glucose homeostasis, pancreatic β-cell preservation, and incretin hormone secretion. However, this duration may not have been sufficient to fully assess the long-term effects of AE, particularly on insulin resistance and metabolic stability. A longer study period could provide deeper insights into potential long-term benefits of AE treatment. Additionally, further investigations are necessary to elucidate the signaling pathways involved in β-cell regeneration, oxidative stress reduction, and inflammation suppression. To provide a definitive conclusion on the effect of AE on SGLT1 expression, a western blot analysis is necessary to quantify protein levels and confirm translational changes following gene expression analysis using RT-PCR. Both techniques will offer a more comprehensive understanding of the gene’s activity by examining both transcription and translation [45].

## Conclusions

This study demonstrated that the AE of nipa palm vinegar effectively ameliorated metabolic abnormalities associated with T2DM in a HFD-STZ-induced rat model. AE treatment, particularly at a dose of 1000 mg/kg, significantly reduced hyperglycemia, improved lipid profiles, and preserved pancreatic β-cell function and hepatocyte morphology. These beneficial effects were attributed to the restoration of insulin secretion, partially mediated by the insulinotropic action of incretin hormones. Further analysis revealed that the presence of DPP-4 inhibitors and the downregulation of SGLT1 contributed to enhanced incretin hormone secretion. These findings highlight the potential of AE as a functional food for T2DM management.

## Electronic supplementary material

Below is the link to the electronic supplementary material.

Supplementary Material 1

Supplementary Material 2

## Data Availability

All data supporting the findings of this study are available within the paper and its Supplementary Information.
