# Supplementary material for: Effect of nipa palm (Nypa fruticans Wurmb.) vinegar on the incretin hormones and intestinal glucose transporters in type 2 diabetes mellitus rat model
Source: BMC Complement Med Ther. 2025 May 30;25:192. doi: 10.1186/s12906-025-04933-8 (PMC12123729; doi:10.1186/s12906-025-04933-8)
Supplement: Supplementary file 2 — Supplementary Material 2 [file 12906_2025_4933_MOESM2_ESM.docx]

**Supplementary File**

# Effect of Nipa Palm (*Nypa fruticans* Wurmb.) Vinegar on the Incretin Hormones and Intestinal Glucose Transporters in Type 2 Diabetes Mellitus Rat Model

**Nur Izzati Razali^1^, Tri Widyawati^2^, Dwi Rita Anggraini^3^, Lim Vuanghao^1^, Nor Adlin Yusoff^1^**

1 Department of Toxicology, Advanced Medical and Dental Institute, Sains@Bertam, Universiti Sains Malaysia, Penang 13200, Malaysia

2 Department of Pharmacology and Therapeutic, Faculty of Medicine, Universitas Sumatera Utara, Medan 20155, Indonesia

3 Department of Anatomy, Faculty of Medicine, Universitas Sumatera Utara, Medan 20155, Indonesia

* Correspondence: noradlinyusoff@usm.my (Nor Adlin Yusoff); Tel.: +604 562 2423

**Histopathologic Score of Liver**

| **Group** | **Slide (code name)** | | **Score*** | | | | | | | | | | | | | | | | | | | | | **Other observations** | |  |  |
| --- | --- | --- | --- | --- | --- | --- | --- | --- | --- | --- | --- | --- | --- | --- | --- | --- | --- | --- | --- | --- | --- | --- | --- | --- | --- | --- | --- |
|  |  |  | **Steatosis** | | | | | | | **Lobular Inflammation** | | | | | | | | **Ballooning** | | | | | |  |  |  |  |
|  |  |  | **0** | **1** | | **2** | | **3** | | **0** | | **1** | | **2** | | **3** | | **0** | | | **1** | **2** | |  |  |  |  |
| NC | LB1C1R1 | | 0 |  | |  | |  | | 0 | |  | |  | |  | | 0 | | |  |  | |  | |  |  |
| NC | LB1C1R2 | | 0 |  | |  | |  | | 0 | |  | |  | |  | | 0 | | |  |  | | Interstitial hemorrhage minimal | |  |  |
| NC | LB1C1R3 | | 0 |  | |  | |  | | 0 | |  | |  | |  | | 0 | | |  |  | |  | |  |  |
| NC | LB1C1R4 | | 0 |  | |  | |  | | 0 | |  | |  | |  | | 0 | | |  |  | |  | |  |  |
| NC | LB3C1R1 | | 0 |  | |  | |  | | 0 | |  | |  | |  | | 0 | | |  |  | |  | |  |  |
| NC | LB3C1R2 | | 0 |  | |  | |  | | 0 | |  | |  | |  | | 0 | | |  |  | |  | |  |  |
| DC | LB1C2R2 | |  |  | | 2 | |  | |  | |  | |  | | 3 | |  | | |  | 2 | |  | |  |  |
| DC | LB1C2R3 | |  |  | |  | | 3 | |  | |  | |  | | 3 | |  | | |  | 2 | |  | |  |  |
| DC | LB1C2R4 | |  |  | |  | | 3 | |  | | 1 | |  | |  | |  | | |  | 2 | | Congestive vein (+) (+) | |  |  |
| DC | LB2C1R1 | |  |  | | 2 | |  | |  | | 1 | |  | |  | |  | | |  | 2 | |  | |  |  |
| DC | LB2C1R3 | |  |  | |  | | 3 | |  | | 1 | |  | |  | |  | | |  | 2 | | Congestive vein (+) | |  |  |
| DC | LB3C3R4 | |  |  | |  | | 3 | |  | |  | | 2 | |  | |  | | |  | 2 | | Congestive vein (+)(+) (+) | |  |  |
| PHZ | LB1C3R1 | |  |  | |  | | 3 | |  | |  | | 2 | |  | |  | | |  | 2 | | Congestive vein (+), necrosis (+) | |  |  |
| PHZ | LB1C3R2 | |  |  | | 2 | |  | |  | |  | | 2 | |  | |  | | |  | 2 | |  | |  |  |
| PHZ | LB1C3R3 | |  |  | | 2 | |  | |  | |  | | 2 | |  | |  | | |  | 2 | | Congestive vein (+)(+) (+), necrosis (+) | |  |  |
| PHZ | LB2C2R3 | |  | 1 | |  | |  | |  | |  | |  | | 3 | |  | | |  | 2 | | Necrosis (+) | |  |  |
| PHZ | LB2C2R4 | |  |  | |  | | 3 | |  | |  | | 2 | |  | |  | | |  | 2 | |  | |  |  |
| PHZ | LB3C2R3 | |  |  | | 2 | |  | |  | |  | |  | | 3 | |  | | |  | 2 | |  | |  |  |
| AE1000 | LB1C4R2 | |  |  | | 2 | |  | |  | |  | |  | | 3 | |  | | |  | 2 | | Congestive vein (+), necrosis minimal | |  |  |
| AE1000 | LB1C4R3 | |  |  | | 2 | |  | |  | |  | |  | | 3 | |  | | |  | 2 | | Congestive vein (+)(+) | |  |  |
| AE1000 | LB2C3R2 | |  | 1 | |  | |  | |  | |  | | 2 | |  | |  | | |  | 2 | | Congestive vein (+) | |  |  |
| AE1000 | LB2C3R4 | |  | 1 | |  | |  | |  | |  | | 2 | |  | |  | | |  | 2 | | Congestive vein (+), necrosis (+) | |  |  |
| AE1000 | LB2C3R5 | |  | 1 | |  | |  | |  | |  | | 2 | |  | |  | | |  | 2 | | Congestive vein (+)(+), necrosis (+) | |  |  |
| **Groups** | **Slide** | **Score*** | | | | | | | | | | | | | | | | | | | | | | | **Other observations** | |  |
|  |  | **Steatosis** | | | | | | | | | **Lobular Inflammation** | | | | | | | | **Ballooning** | | | | | |  |  |  |
|  |  | **0** | | | **1** | | **2** | | **3** | | **0** | | **1** | | **2** | | **3** | | **0** | **1** | | | **2** | |  |  |  |
| AE1000 | LB3C2R2 |  | | | 1 | |  | |  | |  | |  | | 2 | |  | |  |  | | | 2 | |  | |  |
| AE500 | LB1C5R3 |  | | |  | |  | | 3 | |  | | 1 | |  | |  | |  | 1 | | |  | | Congestive vein (+) | |  |
| AE500 | LB1C5R4 |  | | |  | | 2 | |  | |  | | 1 | |  | |  | |  | 1 | | |  | | Congestive vein (+) | |  |
| AE500 | LB1C5R5 |  | | |  | | 2 | |  | |  | | 1 | |  | |  | |  | 1 | | |  | | Congestive vein (+)(+) | |  |
| AE500 | LB2C4R2 |  | | | 1 | |  | |  | |  | | 1 | |  | |  | |  | 1 | | |  | | Congestive vein (+), sinusoid (+)(+) | |  |
| AE500 | LB3C3R1 |  | | |  | | 2 | |  | |  | |  | | 2 | |  | |  | 1 | | |  | | Congestive vein (+), interstitial hemorrhage | |  |
| AE500 | LB3C3R2 |  | | | 1 | |  | |  | |  | | 1 | |  | |  | |  | 1 | | |  | | Congestive vein (+) (+), sinusoid (+) | |  |
| AE250 | LB1C6R3 |  | | | 1 | |  | |  | |  | | 1 | |  | |  | |  | 1 | | |  | |  | |  |
| AE250 | LB1C6R4 |  | | | 1 | |  | |  | |  | | 1 | |  | |  | |  | 1 | | |  | |  | |  |
| AE250 | LB2C5R1 |  | | | 1 | |  | |  | |  | | 1 | |  | |  | |  | 1 | | |  | | Sinusoid (+) | | |
| AE250 | LB2C5R2 |  | | | 1 | |  | |  | |  | | 1 | |  | |  | |  | 1 | | |  | | Sinusoid (+) | | |
| AE250 | LB2C5R3 |  | | | 1 | |  | |  | |  | | 1 | |  | |  | |  | 1 | | |  | |  | | |
| AE250 | LB3C2R1 |  | | | 1 | |  | |  | |  | | 1 | |  | |  | |  | 1 | | |  | | Sinusoid (+) | | |

*** Score based on adapted Non-Alcoholic Fatty Liver Disease (NAFLD) Activity Score (NAS)**

**Non-Alcoholic Fatty Liver Disease (NAFLD) Activity Score (NAS)**

| **Feature** | **Points** | **Criteria** |
| --- | --- | --- |
| A. Steatosis | 0 | <5% |
|  | 1 | 5 to 33% |
|  | 2 | >33 to 66% |
|  | 3 | >66% |
| B. Lobular inflammation | 0 | None |
|  | 1 | <2 foci per 200X field |
|  | 2 | 2 to 4 foci per 200X field |
|  | 3 | >4 foci per 200X field |
| C. Ballooning | 0 | None |
|  | 1 | Few ballooning cells |
|  | 2 | Many cells/prominent ballooning |
